# Configurable IoT Open-Source Hardware and Software I-V Curve Tracer for Photovoltaic Generators

**DOI:** 10.3390/s21227650

**Published:** 2021-11-18

**Authors:** Isaías González, José María Portalo, Antonio José Calderón

**Affiliations:** Department of Electrical Engineering, Electronics and Automation, University of Extremadura, Avenida de Elvas, s/n, 06006 Badajoz, Spain; jportalo@alumnos.unex.es (J.M.P.); ajcalde@unex.es (A.J.C.)

**Keywords:** IoT, renewable energy sources, photovoltaic energy, I-V curve, monitoring and data acquisition, microgrid, open-source, communication protocols

## Abstract

Photovoltaic (PV) energy is a renewable energy resource which is being widely integrated in intelligent power grids, smart grids, and microgrids. To characterize and monitor the behavior of PV modules, current-voltage (I-V) curves are essential. In this regard, Internet of Things (IoT) technologies provide versatile and powerful tools, constituting a modern trend in the design of sensing and data acquisition systems for I-V curve tracing. This paper presents a novel I-V curve tracer based on IoT open-source hardware and software. Namely, a Raspberry Pi microcomputer composes the hardware level, whilst the applied software comprises mariaDB, Python, and Grafana. All the tasks required for curve tracing are automated: load sweep, data acquisition, data storage, communications, and real-time visualization. Modern and legacy communication protocols are handled for seamless data exchange with a programmable logic controller and a programmable load. The development of the system is expounded, and experimental results are reported to prove the suitability and validity of the proposal. In particular, I-V curve tracing of a monocrystalline PV generator under real operating conditions is successfully conducted.

## 1. Introduction

Photovoltaic (PV) technology is one of the most widespread renewable energy sources (RES) [1,2] and contributes to reducing greenhouse gas emissions and fighting against climate change [3]. In intelligent energy facilities conceived under the paradigm of smart grids and microgrids, PV generators are commonly the main source of renewable energy [4]. In these facilities, PV can be combined with other equipment for energy production and consumption such as wind turbines, batteries, and hydrogen-related devices (fuel cells and electrolyzers).

In PV-based grids, it is required to monitor the state and operation of the PV devices. In this regard, the efficiency of PV cells under natural conditions is measured using current-voltage (I-V) characteristic curves [5]. Consequently, to evaluate the performance of PV modules, it is necessary to measure their I-V output characteristics [6].

I-V curves are obtained by performing a voltage sweep on the PV module, while measuring the output current which is delivered to a connected load [5]. Such curves display maximum voltage and current values of a module in a given setting [5]. This way, the analysis of the curves provides direct information on the electrical state of the module, allowing the researcher to obtain data on the expected performance under different conditions of irradiance and load [7]. The measurement system used to acquire data of the PV modules and to visualize the I-V curve is commonly known as the I-V curve tracer. 

In the context of RES, monitoring and data acquisition are essential to recognize the resources available on-site, evaluate electrical conversion efficiency, detect failures, and optimize electrical production [8]. In particular, the characterization of PV modules through I-V curves is required for different purposes, and the applicability of such curves has been widely reported in the literature. For instance, I-V curve tracing is the most commonly applied technique for the electrical characterization of PV modules [9]. An in situ measurement system of PV characteristics can provide valuable information for optimized power generation [10]. I-V curves are widely used to evaluate power generation performance and detect fault conditions of PV generators [11]. 

Aging effects of PV cells affect the I-V curve [10], and the consequent degradation is usually assessed by means of such I-V characteristics [6]. It must be noted that failures in PV modules may be caused by several reasons such as corrosion failures, cell cracks, hot-spots, encapsulation failures, electrical or mechanical connection failures, potential induced degradation, accumulation of dust or soiling, or partial shading, among others [12].

In the context of degradation and failure analyses, detailed parameter fitting can be carried out using the I-V characteristics of the strings or modules for a deeper understanding of degradation mechanisms prior to failure [13]. According to [14], the I-V curve measurement method is time-consuming, but it is very reliable and considered a paramount step in fault detection. For diagnosis, electrical parameters are extracted from the measured I-V curves such as the short circuit current, the open-circuit voltage, the maximum power-point, and the fill factor [6,14]. Indeed, decisions on the replacement of faulty or degraded devices are better taken based on a direct measurement than based on estimation [15].

In addition, another application of I-V curves is related to the modeling of PV modules, which requires estimating or measuring certain parameters. In this regard, a data-acquisition system is essential to collect and store I-V curves so simulated I-V curves can be plotted based on different models [16]. Namely, the well-known single diode model (SDM) requires estimating the values of series and parallel resistances which can be calculated from the I-V curves [17]. 

However, the manufacturer provides curves measured at laboratory conditions, obtained under standard conditions of temperature and irradiance (standard test conditions, STC), which do not correspond to real operation in physical facilities. These differences affect the I-V characteristic and, thus, the module production, so it is crucial to use an I-V curve tracer [6].

The relevance of I-V tracing can be witnessed in the literature; the instrumentation and monitoring equipment for such a task have received important research efforts. For example, in-depth reviews of curve tracers according to their topologies can be found in [7,11]. Additionally, diverse equipment is available in the market and in the scientific literature. On the one hand, commercial curve tracers can be found in the market, such as in [18,19,20]. Their main advantage is the reliable measurements that are performed, guaranteed by the manufacturer of the device. On the contrary, the most noticeable drawbacks are that commercial systems are generally expensive and closed for modifications [8]. Furthermore, another important disadvantage is that the control software of commercial tracers is not prepared for an automatic experimental campaign of measurements [21].

On the other hand, custom-designed I-V curve tracers constitute an important trend in recent years. Diverse works have designed curve tracers using general purpose electronic boards such as microcontrollers and digital signal processors (DSP). For example, in [22,23], a portable I-V curve tracer is based on a DSP, being connected to a personal computer (PC) through serial communication. Vega et al. [6] combine a peripheral interface controller (PIC) and an electronic load (MOSFET) to implement an I-V curve tracer. Acquired data are stored on a PC or a smartphone. A low-cost PIC is used by Ortega et al. [12] to develop a prototype of an I-V curve tracer for individual modules in large photovoltaic systems. A curve tracer developed around a commercial low-cost embedded microcontroller (TivaC) is presented in [24]. Additionally, a low-cost microcontroller is the core of the curve tracer presented in [15], where measurements are stored in local memory and downloaded through a universal synchronous asynchronous receiver transmitter (USART) connected to a Bluetooth device.

Moreover, within custom-designed curve tracers, new developments are progressively including Internet of Things (IoT) open-source technology. Namely, open-source hardware platforms such as Arduino and Raspberry Pi (RPi) are being introduced in research projects and facilities. In the scope of PV energy, these devices are applied for data acquisition and monitoring tasks. For example, Arduino is used in [25,26] to sense the temperature of PV modules. With higher computation capabilities, the RPi microprocessor is used to implement monitoring systems for PV-based microgrids in [4,27,28] and for PV plants in [5,29,30].

In particular, to deploy I-V curve tracers, there are still scarce developments involving such IoT open-source technology, so the most recent research works will now be discussed. Within the open-source community, there is information publicly available about I-V curve tracers using Python and the Arduino microcontroller [31]. In the curve tracer proposed in [21], Arduino is responsible for managing a capacitive load, while data storage and visualization are performed by a PC. Arduino is used together with a commercial data logger in [32] to handle a MOSFET load in order to trace I-V curves of PV modules. An Arduino together with a PC is used in [33] to deploy an I-V curve tracer, the PC acting as a storage means for the measured data. The work reported in [34] applies an Arduino board with data storage on an SD card to collect the data of PV modules under shading conditions. Papageorgas et al. [10] develop a low-cost curve tracer involving an open-source platform with an embedded microcontroller called Polytropon and message queueing telemetry transport (MQTT) as a communication protocol.

Regarding the use of RPi, the following works are found. In [35], an IoT-based remote I-V tracing system is developed using an RPi and a cloud-based server aimed at analyzing soiling losses in distributed solar facilities. An RPi is used in [36] to implement a plug and play I-V curve tracer oriented toward the diagnosis of PV modules. A power MOSFET transistor is used as the electronic load during characterization, the data being recorded on the RPi and on an intermediate file transfer protocol (FTP) server. In [37], an RPi is used as the main component in a so-called outdoor test facility (OTF) with IoT capabilities employed to capture I-V and P-V curves of PV modules. Python scripts are used, and experimental validation is reported.

Some relevant requirements and trends of curve tracers have been identified in the previous literature. For example, in [33], it is pointed out that the measure of the entire I-V curve in a short time requires a suitable data acquisition device. Reference [7] identifies various trends in the advancement of curve tracers, among which low-cost measurement systems and low-cost communications are highlighted. In the same sense, the important role that reliable low-cost communications play is emphasized in [15]. 

The utilization of open-source and IoT technologies for curve tracing and monitoring constitutes another new trend [16,38]. Furthermore, such technologies encourage the previously mentioned trends of low-cost measurements and communication systems. These technologies provide rapid development and cost-effective solutions for smart monitoring systems [16]. Related to costs, as pointed out in [24], the low-cost characteristic of open-source platforms provides greater accessibility to I-V curve tracing equipment for any research or academic center. 

An issue of the existing literature is signaled in [38]: there are papers that do not offer information either about the measurement performance or about the equipment used, showing only the results. Moreover, as it is asserted in [7], most of the curve tracers found in the literature are complex and difficult to integrate in real scenarios. 

Aiming at overcoming the aforesaid drawbacks and integrating the identified trends, this paper presents the development of a novel IoT open-source hardware and software I-V curve tracer to characterize PV generators. The RPi microcomputer composes the hardware level; concerning software, Python, MariaDB, and Grafana are applied for data acquisition, storage, and visualization. Open communication protocols, such as Modbus TCP, enable seamless data exchange with proprietary equipment. The program coded in Python is responsible for automating the curve tracing of the PV modules through the modification of the current demanded by an electronic programmable load. The developed system is oriented towards the I-V curve tracing for already existent functioning facilities that require diagnostics, analyses, and/or modeling. In such a situation, this curve tracer is coupled to the facility by means of open protocols and shares data without altering the installation.

For the sake of clarity, a table summarizing the aforementioned literature as well as the present proposal has been elaborated (Table 1). The considered categories are the following: Device, referred to as the equipment used to collect data from PV modules; Load, to indicate the type of load; Data storage means, to discriminate if local (within the Device) or remote accumulation is applied; Language, for a clear identification of the programming language used to gather data; and Communication, in order to specify the protocols for data sharing. In the cases where the information has not been found, Unspecified has been written. 

In view of the previous table, it can be derived that the presented curve tracer is a novelty due to the fact that it is a unique proposal which combines open-source components (hardware and software) and open communication protocols, all being managed by the RPi. In other words, in most of the literature, an IoT open-source device is used to gather data from sensors and handle the load, data storage and visualization being performed by external equipment or services such as PCs or cloud servers. The present work is the only proposal where the RPi is responsible for automating all the tasks involved in the I-V tracing: load sweep, data acquisition, data storage, communications, and visualization in real-time.

Moreover, as can be observed in Table 1, some works do not report information about certain aspects such as the programming language or data storage means (software or hardware), as it has been previously indicated in [38]. Moreover, none of the surveyed references apply a programmable electronic load to perform the I-V tracing. Resistive, capacitive, or electronic loads (power MOSFET) are among the common methods [7], but they require designing specific electronic circuitry for the curve tracing process and only serve for such a purpose. On the contrary, programmable loads are commonly used in microgrids and PV facilities [4,20,39,40,41] to emulate the behavior of DC or AC loads in order to test control algorithms and energy management strategies under different load profiles. In this regard, the target groups of this paper are scientists and practitioners in the scope of PV-based microgrids and facilities involved in research and development (R&D) activities.

In addition, the validation of the proposal is performed with a medium-scale PV generator under real conditions, which constitutes a requirement to demonstrate the suitability of open-source technologies [4]. 

It must be remarked that the presented curve tracer is used to characterize and diagnose a PV generator integrated in a smart microgrid (SMG) which combines renewable sources with hydrogen. Such a facility is framed in an R&D project envisioned to develop a digital replica of the subsystems of the microgrid. 

The main contributions of the work are now summarized:

- Open-source hardware and software is applied for data storage and visualization. As a consequence, easy and low-cost deployment and replication are feasible;
- Open communication protocols are used to provide a seamless data exchange;
- The curve tracer can be coupled to an already existent PV generator and gather operational data;
- Experimental results achieved on a medium-scale PV generator, not only for a laboratory scale, are reported to prove the suitability of the developed curve tracer;
- Capability of configuration for facilities with a larger number of sensors to manage as well as different communication protocols;
- Utilization of proprietary medium-scale programmable electronic load providing accurate and reliable measurements;
- IoT-enabled remote monitoring of real-time data in the form of time-series;
- Automated data acquisition under programmed conditions without requiring operator intervention.

The structure of the rest of the paper is as follows. The Section 2 describes the developed I-V curve tracer concerning hardware, software, and communications. Section 3 deals with the achieved results from a PV generator of 1100 W, whereas the associated discussion is carried out in Section 4. Finally, the main conclusions of the reported work and further research guidelines are addressed.

## 2. Developed I-V Curve Tracer 

**Figure 1:**
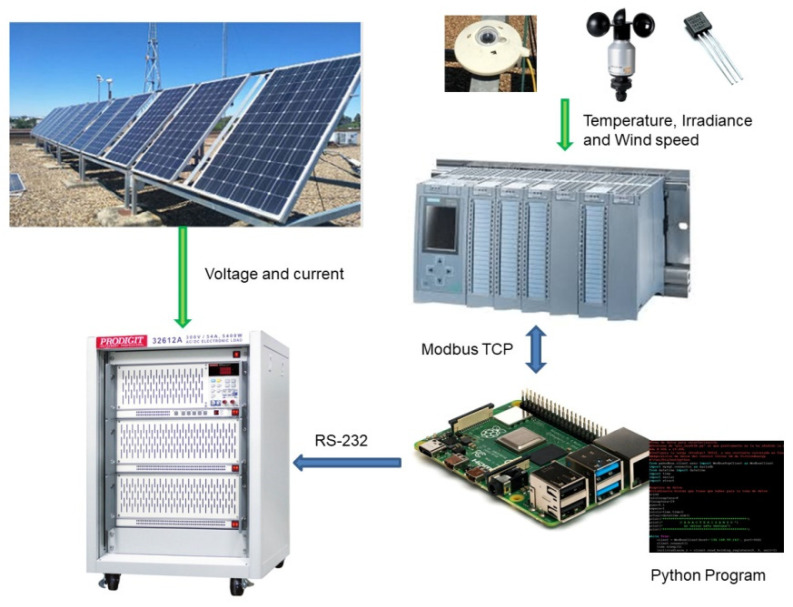
Interconnection of the I-V curve tracer components together with equipment of the existing microgrid.

The developed curve tracer is solved by a software application made in Python and executed on an RPi, as well as a database and a data visualization interface. The version of the microcomputer is the RPi 3 model B+. As commented in the previous section, the proposed curve tracer is applied to an existing SMG equipped with an automation system based on a Programmable Logic Controller (PLC) model S7_1516, which is in charge of the energy management of the SMG. Figure 1 shows the interconnection of all the devices used in the I-V curve tracer. This figure shows the sensors involved (irradiance, voltage, current) together with the PLC, the RPi, and a programmable electronic load. The proposed platform takes advantage of an electronic programmable load model Prodigit 32612A (New Taipei City, Taiwan). This legacy device accepts communication through an RS232 interface in order to exchange commands and is used to configure the current profiles demanded by the photovoltaic panels.

**Figure 2:**
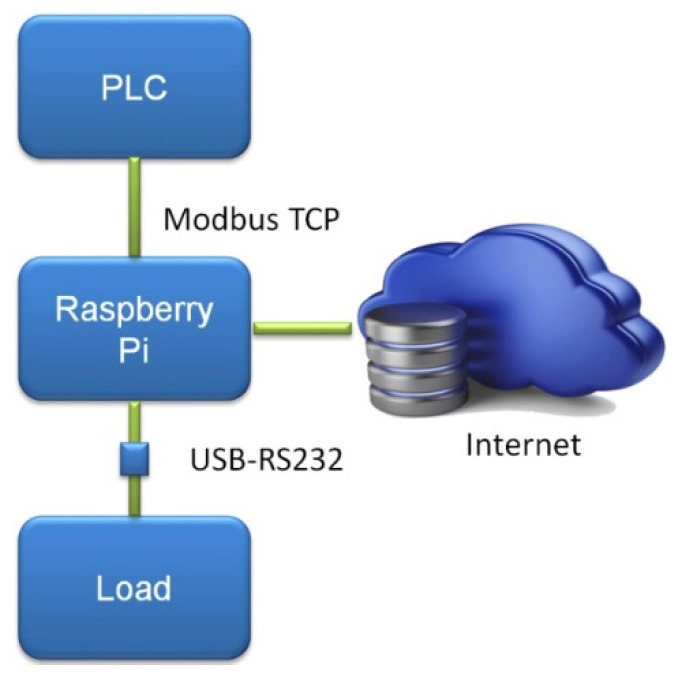
Communications diagram of the I-V curve tracer.

The communications diagram of the deployed system can be seen in Figure 2. The RPi acts as a Grafana server, so the user/operator can visualize and download the data processed by the curve tracer through a web browser running on a computer or smartphone connected to the Internet. Namely, the Grafana software provides a user-friendly graphical user interface (GUI) for real-time access to numerical and graphical information about the measurements of the PV system during the tracing. 

Among other elements, the RPi includes serial communication through universal serial bus (USB) ports, so a protocol converter from USB to RS232 has been required to establish communication between the load and the RPi. 

Concerning sensors, Table 2 summarizes the magnitudes that are measured and the corresponding sensor. It must be noted that the required sensors could be connected to the RPi in a direct manner or through proper electronic boards. In this sense, the presented solution is applicable to already existing automation and monitoring systems or for new facilities without such systems. Using open protocols, such as Modbus TCP, enables easy communication given the widespread availability of this protocol in automation and energy-related equipment [28]. In addition, Modbus TCP has been pointed out as an industrial IoT communication protocol [42] and is supported by both open-source and proprietary equipment.

**Figure 3:**
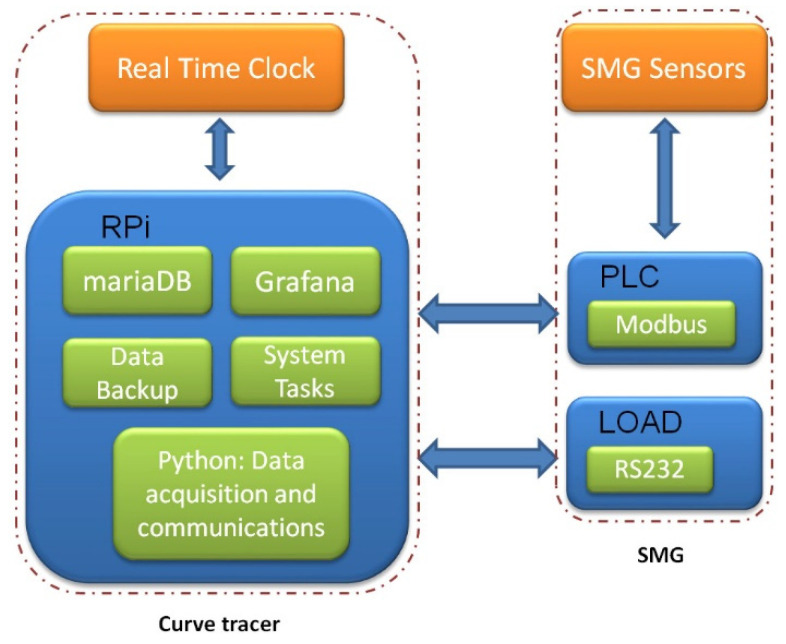
Functionalities implemented by the curve tracer.

A block diagram with the functionalities associated with each component is illustrated in Figure 3. From a functional viewpoint, it is interesting to note that all the tasks required for the process of I-V curve tracing rely on the RPi. On the one hand, this microprocessor acquires data from the PLC and stores them in a database (mariaDB). Data backup is also handled by the RPi together with other system tasks. On the other hand, the programmable load is managed through Python commands. Lastly, the Grafana server is hosted to establish Internet-enabled data visualization in real-time.

**Figure 4:**
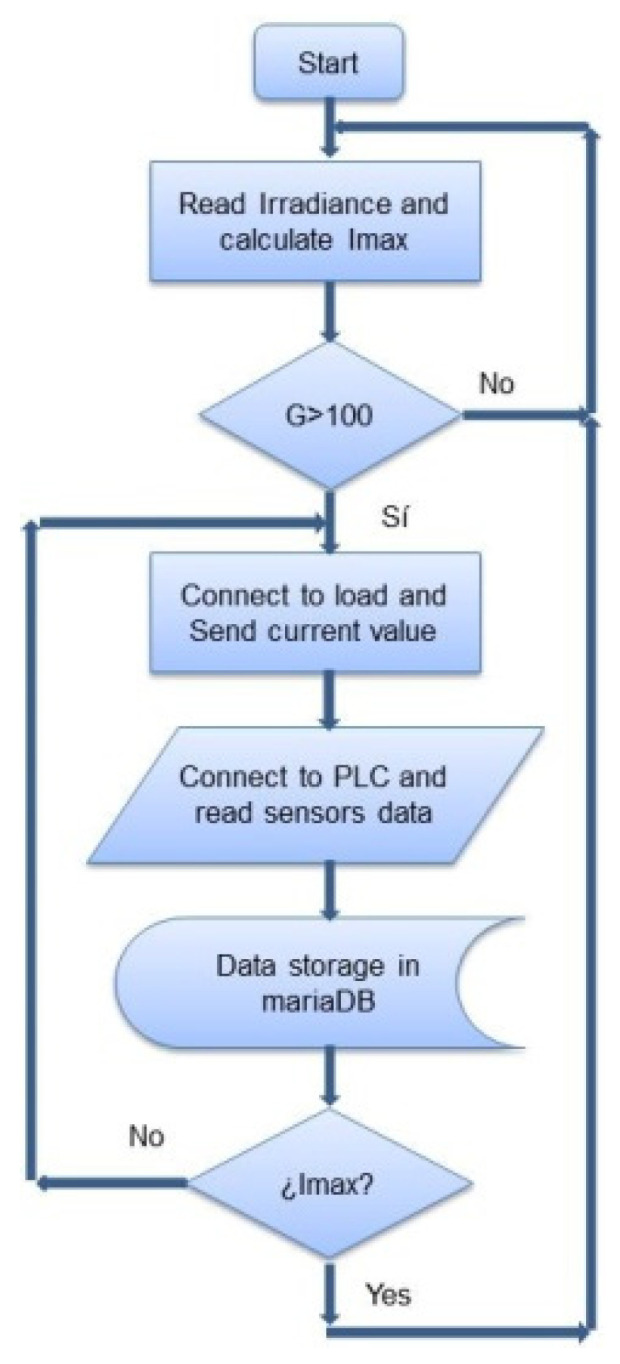
Flowchart of operations performed by the curve tracer.

Figure 4 shows the flow diagram of the algorithm that implements the I-V tracer. First of all, the irradiance existing at a given time is measured. It is verified whether the measured irradiance exceeds the preset threshold of 100 W/m^2^ to initiate the current profile generation and data acquisition processes. Once the minimum irradiance condition is met, a profile of the current demanded by the electronic load is created, which progressively increases from zero to the maximum possible current that the PV module(s) can deliver for the sensed irradiance. The maximum value is calculated based on the existing modules configuration and the irradiance at each moment. This ensures that the PV generator will not be required to provide currents that cannot be achieved for this irradiance value. In particular, the following equation has been used to determine the maximum current, Imax:

Imax = np × (0.0055 × G + 0.1),(1)

where np is the number of paired modules, and G is the incident irradiance on the plane of the modules.

**Figure 5:**
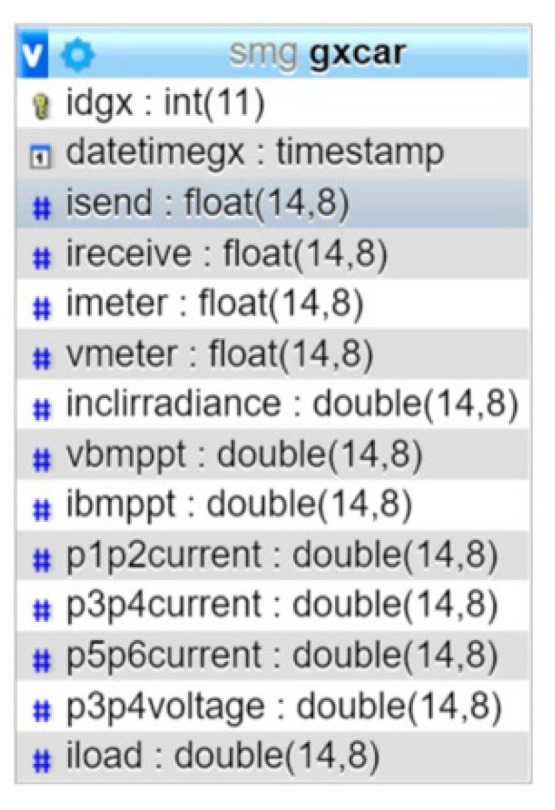
Database created with mariaDB for characterization of PV modules.

The following step consists of establishing communication via RS232 from the RPi to the programmable electronic load, so the current value corresponding to each instant is sent. Next, the RPi takes the sensor data obtained by the PLC through a Modbus TCP channel, as well as from the electronic load itself through the RS232 connection. After this, the retrieved data are stored in a mariaDB database specifically designed for this purpose (Figure 5). This process is carried out continuously for each PV module current until the maximum current set is reached. 

**Figure 6:**
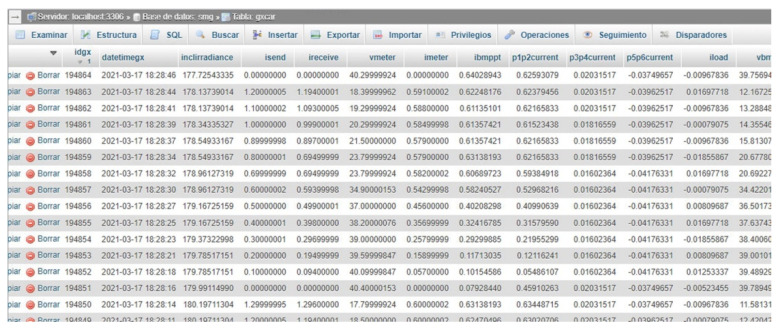
Data taken during characterization of PV modules.

In this way, all the necessary data for the characterization of the photovoltaic panels are acquired and stored. As a sample, Figure 6 shows the data taken for the characterization of the PV modules.

To achieve representative data, the irradiance should change as little as possible during the characterization. Each acquisition cycle can vary from 24 s, for an irradiance of 200 W/m^2^ and a single panel configuration (12 samples), to 320 s, considering an irradiance of 1000 W/m^2^ for the whole group of panels (320 samples), keeping the step sampling rate at 0.1 A. During these short intervals, irradiance is scarcely altered. 

**Figure 7:**
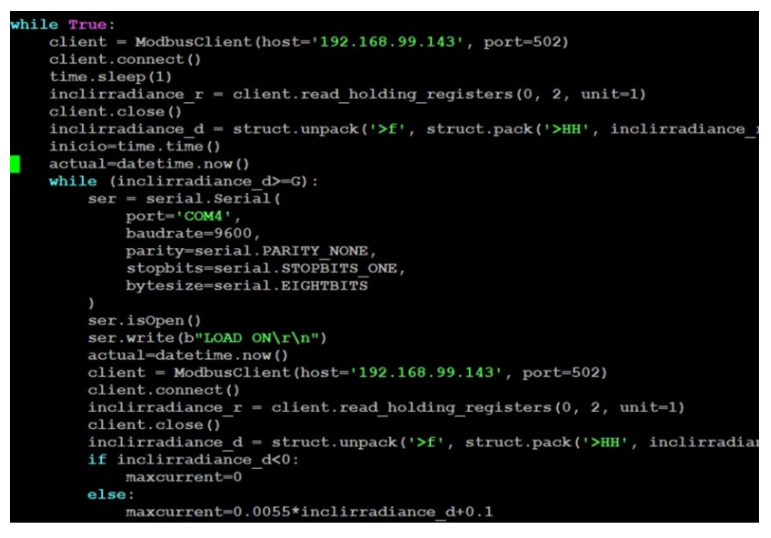
Python code for I-V curve tracing.

On the other hand, Figure 7 shows a screenshot of part of the Python code running to automate the labeled stages and, hence, the I-V curve tracing.

Aiming to illustrate the described sequence, the main code of Python concerning the load management is shown in Algorithm 1. To begin with, an instance for communication is created, specifying parameters such as port, transmission bit rate, parity bit, etc. After that, the connection is open, and the commands to determine the load current are sent. Namely, the load is activated, the operation mode is selected, and the current demanded to the PV module(s) is established. Moreover, the reached voltage and current values are retrieved. Finally, the connection is closed.
**Algorithm 1** RS232 Communication through Python1: **while** (inclirradiance_d>=G): 2:  **ser** = serial.Serial( 3:   **port** = ‘COM4’, 4:   **baudrate** = 9600, 5:   **parity** = serial.PARITY_NONE, 6:   **stopbits** = serial.STOPBITS_ONE, 7:   **bytesize** = serial.EIGHTBITS 8:  ) 9:  **ser.isOpen()** 10:  **ser.write**(b”LOAD ON\r\n”) 11:… 12:   **cadena** = “LIN:A “+str(i/10)[0:5]+”;\r\n” 13:   **ser.write**(cadena.encode()) 14:   **ser.write**(b”LIN:A?\r\n”) 15:   **out** = ser.readline() 16:   **ser.write**(b”MEAS:CURR?\r\n”) 17:   **out1** = ser.readline() 18:   **ser.write**(b”MEAS:VOLT?\r\n”) 19:   **out2** = ser.readline() 20:… 21:  **ser.close()**

## 3. Results

In this section, the experimental results of applying the I-V curve tracer are reported to demonstrate its successful operation. Namely, a PV generator hybridized with hydrogen in a stand-alone SMG, placed at the University of Extremadura (Spain), is fully characterized. 

### 3.1. Experimental Setup

**Figure 8:**
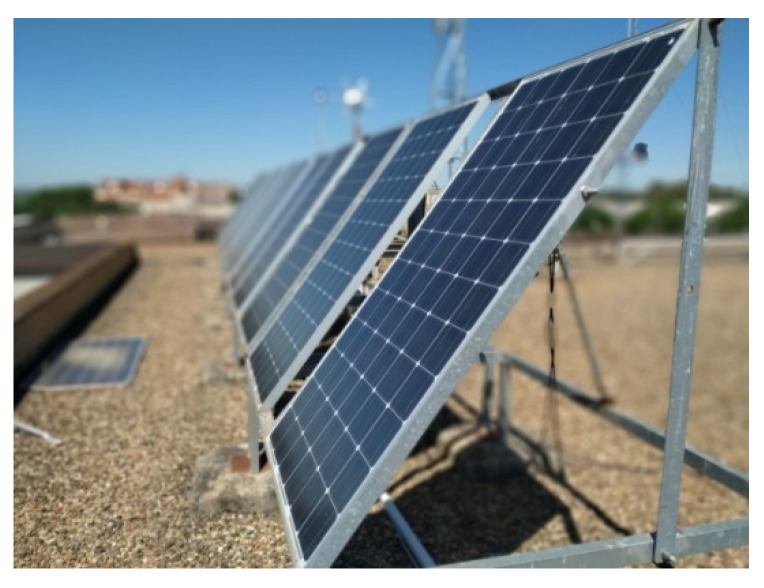
PV generator for experimentation.

The PV generator (Figure 8) consists of six monocrystalline modules, each one with maximum output of 185 W, providing a total power of 1110 W. These modules have a fixed inclination angle, the irradiance measured being in the same plane. The main parameters of the PV modules are listed in Table 3. Note that electric characteristics are given for STC by the manufacturer.

**Figure 9:**
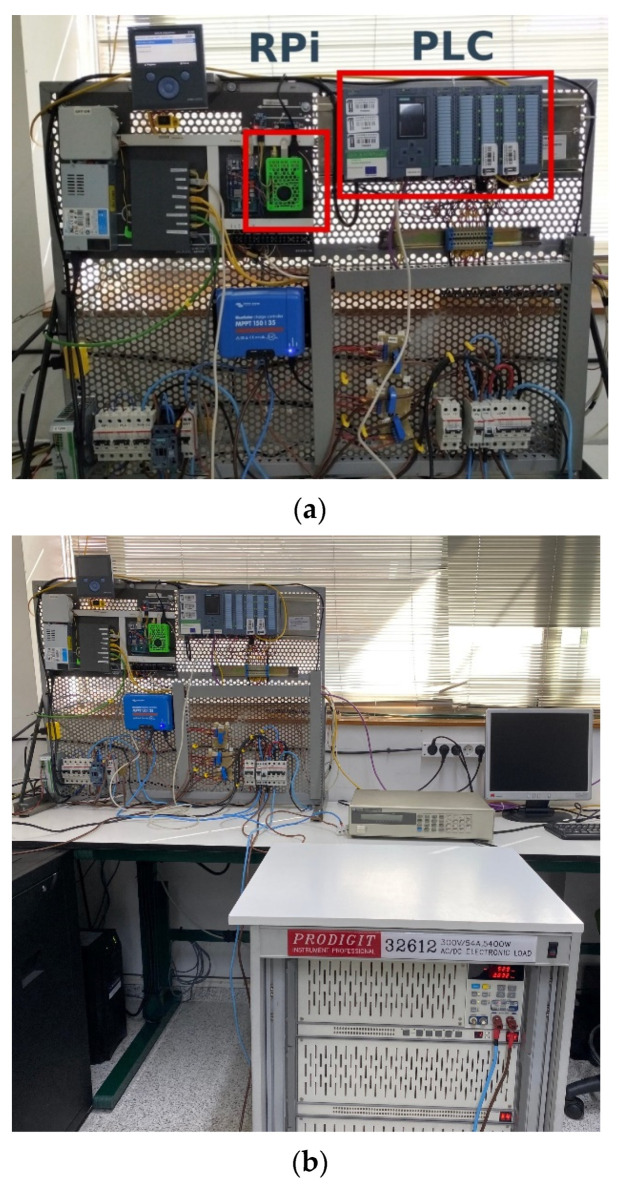
Setup in laboratory: (**a**) detailed view of curve tracer; (**b**) entire view including the programmable load.

**Figure 10:**
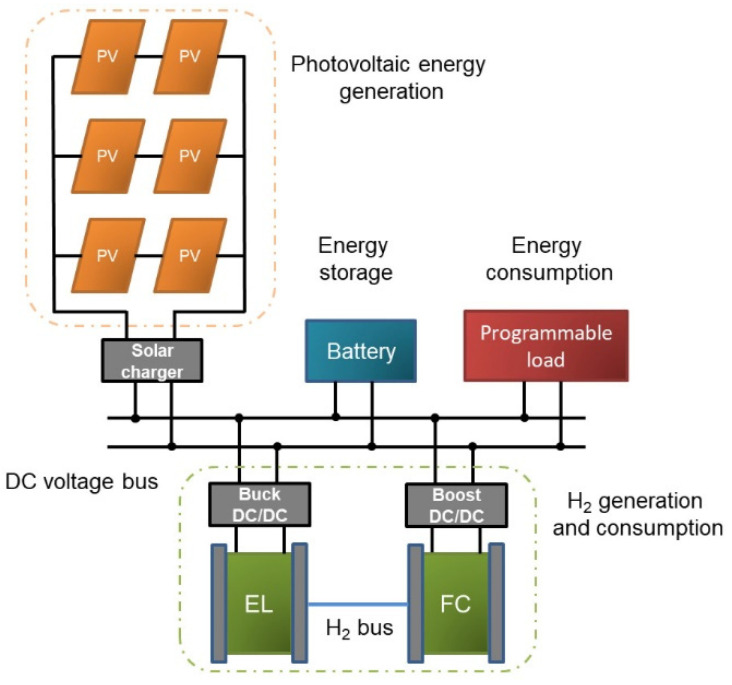
Block diagram of SMG where the PV modules are installed.

The curve tracer is coupled to the PLC and the load of the SMG in the laboratory as can be observed in Figure 9a. Note that an Ethernet switch allows data exchange between the RPi and the PLC. The programmable load can be seen in Figure 9b, also placed in the laboratory setup. On the other hand, the block diagram of the SMG is depicted in Figure 10. As can be observed, the PV array is linked to a DC voltage bus through a solar charger. A battery acts as electrochemical energy storage whilst the programmable load conducts the energy consumer role. Regarding hydrogen generation and consumption, an electrolyzer (EL) produces hydrogen, harnessing the surplus of PV energy, and a fuel cell (FC) performs the opposite process, converting hydrogen into electricity when there is no renewable energy availability. A more detailed description of the SMG components can be found in [4,28].

### 3.2. Data Visualization

The measurement process was carried out during different days due to the variability of weather conditions (cloudy and rainy days, etc.). More than 194,000 samples were recorded during the whole measurement campaign. The stored data are represented through the GUI created in Grafana, which displays the involved magnitudes in the form of time-series. 

**Figure 11:**
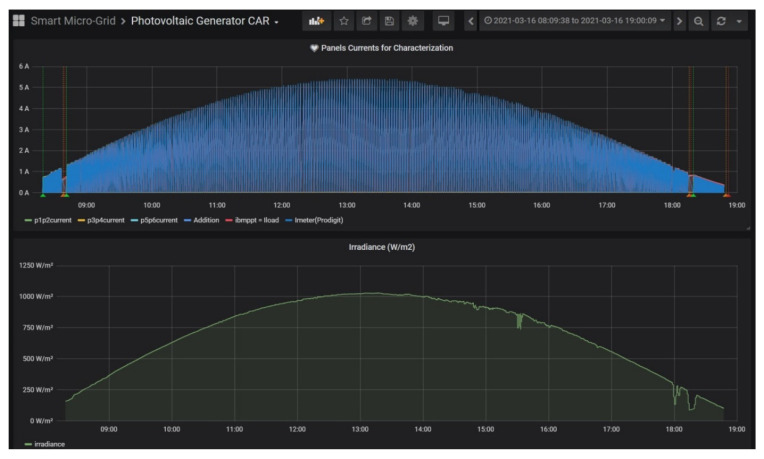
Grafana GUI displaying time-series of PV current and irradiance during a day of characterization.

As a proof of the visualization capabilities, Figure 11 depicts the aspect of the GUI showing the measurements during a day of the PV generator characterization, in particular, 16 March 2021 from 8:00 to 19:00. The typical curve of solar irradiance along the day can be appreciated in the lower graph, reaching a maximum value of 1031.48 W/m^2^ at 13:17. During this day, the procedure begins at 8:18 once the irradiance threshold of 100 W/m^2^ is exceeded and lasts until 18:47. The top chart represents the current delivered by the PV generator, which fluctuates according to the management performed by the Python program of the curve tracer.

**Figure 12:**
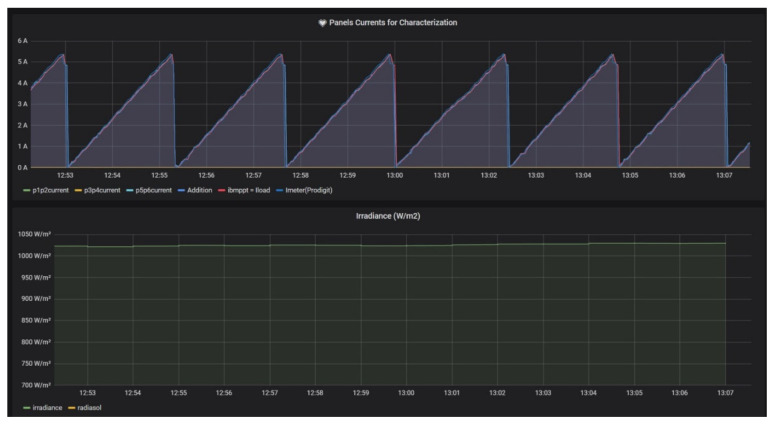
Detailed view of Grafana GUI to observe PV current and irradiance during characterization.

Figure 12 contains a detailed view of the GUI during the same day for a better observation of the magnitude evolution. In the top graph, it can be seen that the current delivered by the PV generator (blue color) and the load current (red color) are coincident and both exhibit a saw tooth-shaped evolution, coherent with the implemented algorithm. The sensed irradiance during the viewed interval is 1027 W/m^2^.

**Figure 13:**
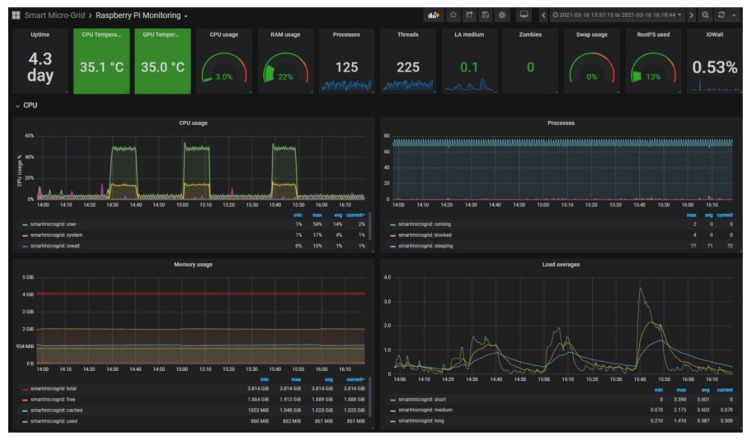
Dashboard devoted to monitoring the resources of RPi during experiments.

In order to verify the capabilities of the curve tracer, the computational resources of the RPi are also monitored by means of Grafana. To this aim, the GUI includes a dashboard based on Telegraf [43] to visualize the central processing unit (CPU) temperature and load, memory usage, and network statistics. Figure 13 shows this dashboard during the characterization experiments on the same day shown in the previous figures. There are some relevant aspects to discuss in this sense. The usage of CPU is observable in the graph placed in the top left position, and its nominal value is around 4%. There are certain intervals during which the usage rises up to 17% for the system (yellow color) and up to 54% for the user (green color), respectively. These increments are due to Grafana operations, e.g., access for online monitoring and requests to the database. Another parameter is the memory usage (graph in the low and left part) where less than 1 GB is used (yellow line) and around 1 GB is cached (blue line), leaving 2 GB free (orange line), showing a stable behavior. Concerning the CPU temperature, it has a stable value around 35 °C, being an appropriate level to avoid overheating issues. 

### 3.3. I-V Curves of PV Generator

To achieve a proper validation, I-V curves have been obtained under real operating conditions for the PV generator. In addition, three configurations of the modules have been applied: a single module, a pair of modules connected in series, and the whole generator, consisting of the parallel connection of three pairs.

For the curve tracing, it has been required to select the data for the different irradiances close to the values commonly provided by manufacturers and reported in the literature, namely 200 W/m^2^, 400 W/m^2^, 600 W/m^2^, 800 W/m^2^, and 1000 W/m^2^. Due to the short duration of the data acquisition intervals, the initial and final values of irradiance are averaged. Table 4 shows the measurements of the incident irradiance and the temperature of the modules during the characterization campaign for each one of the described electrical configurations. Moreover, electrical parameters of the generator can be measured in the I-V curves, such as short circuit current, open-circuit voltage, fill factor, etc.; hence, in Table 4, such parameters are also included.

**Figure 14:**
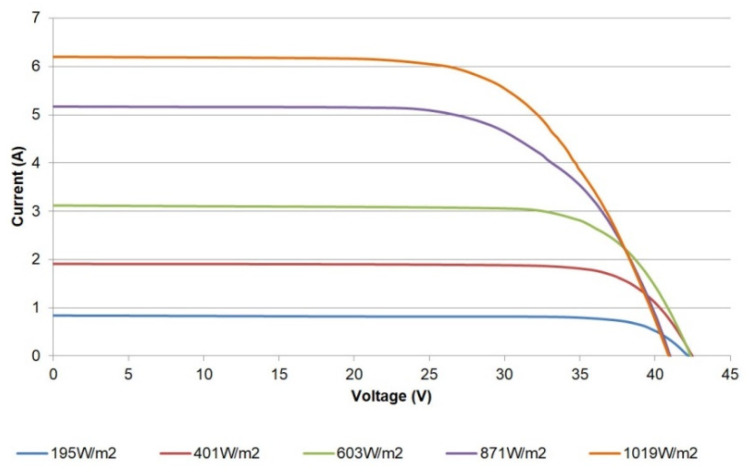
I-V curves for a single PV module.

Figure 14 shows the I-V curves obtained for a single module. The shape and trend of the curves correspond to those expected, matching the information provided by the manufacturer. As can be observed, the open circuit voltage (Voc) decreases whilst the irradiation increases. This effect is due to the associated temperature increase, which causes the curves to move to the left. In particular, the open circuit voltage strongly depends on temperature, while its dependence on irradiance has a modest effect [17]. This relationship can be expressed through Equation (2) [17]:

Voc(T) = Voc,_STC_ + *µ*_Voc_ (T − T_STC_), (2)

where Voc,_STC_ is the open circuit voltage for STC, T_STC_ corresponds to the STC temperature, and *µ*_Voc_ is the voltage temperature coefficient, found in the PV module datasheet. For the LDK Solar 185D-24S, such a coefficient has a value of −0.34%/°C, so it is easy to check that temperature increments give place to decrements of Voc.

**Figure 15:**
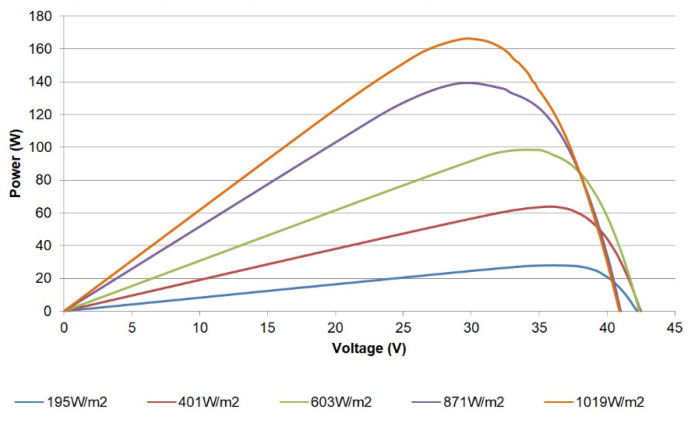
P-V curves for a single PV module.

In a similar sense, the power-voltage (P-V) curve can also be plotted from the acquired data; for instance, Figure 15 shows such a curve for the single PV module. Valuable information such as the maximum power point values (power, current, and voltage) for sensed irradiances can be studied through these curves. 

The maximum power produced (165 W) by the module is lower than that reported by the manufacturer (185 W) given the fact that the existing conditions differ from the STC. Moreover, the degradation of the module also contributes to reducing the peak power that can be delivered.

Following the validation procedure reported in [23,33,34,36,37], the experimental measurements are reproduced by means of a simulator of PV modules based on the SDM. This model is based on the equivalent circuit and is the most widely used method to provide an estimation of the current generated by a PV cell. The circuit consists of a single diode connected in parallel with a photo-generated current source (*I_PH_*), a series resistance (*R_S_*) to represent voltage drops and internal losses, and a shunt resistance (*R_SH_*) to take into account the leakage currents. Equation (3) describes the model for a module of *N_S_* cells in series:

(3) $$I=I_{PH}-I_{o}\left[exp\left(\frac{V+IR_{S}}{nN_{S}V_{TH}}\right)-1\right]-\frac{V+IR_{S}}{R_{SH}}$$

where *I_o_* is the saturation current of the diode, *V* is the output voltage, and *V_TH_* is the thermal equivalent voltage. The last variable is given in terms of the electron charge, *q*; the Boltzmann constant, *K*; the cell temperature, *T*; and the diode ideality factor, *n*, according to Equation (4):

*V_TH_* = *KT*/*q*, (4)

**Figure 16:**
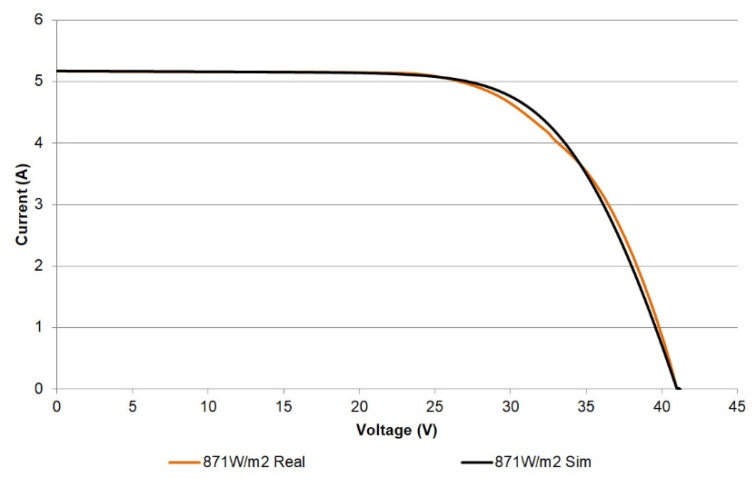
Experimental and simulated I-V curves for a single PV module at 871 W/m^2^.

The I-V curve experimentally measured with the curve tracer at an irradiance of 1019 W/m^2^ and temperature of 45.1 °C is plotted in Figure 16 (black color) together with the curve provided by the SDM simulator (orange color). As can be observed, the curves show the same trend with very scarce differences. Namely, the ideality factor of the SDM explains the difference appreciated in the knee of the curve [36].

**Figure 17:**
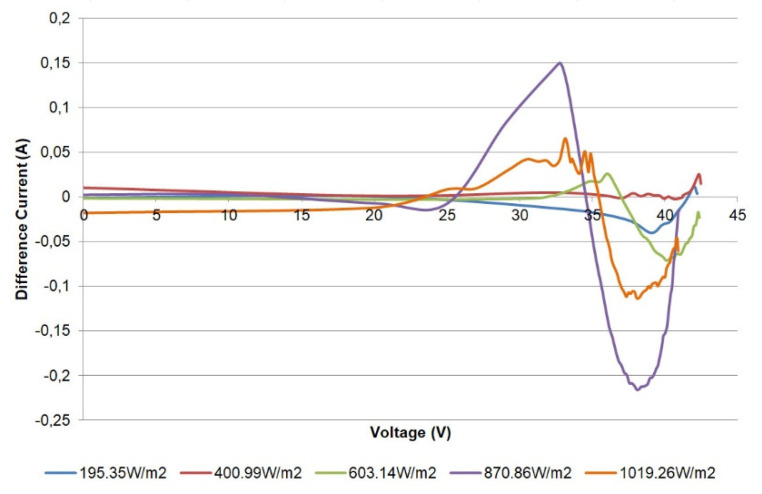
Difference of measured and simulated currents for the single PV module.

For a better appreciation, the difference between the currents (simulated and measured) can be used to illustrate the achieved fitting [44,45]. In this regard, Figure 17 shows the difference of currents for the characterized module versus the voltage at the reported irradiance levels. The errors are small, reaching a maximum value of 0.21 A for 870 W/m^2^. In Figure 14 and Figure 17, it can be seen that the maximum values of these differences are located in a reduced range between the maximum power point (voltage higher than 29 V) and the Voc. These results exhibit proper agreement with the well-known SDM.

**Figure 18:**
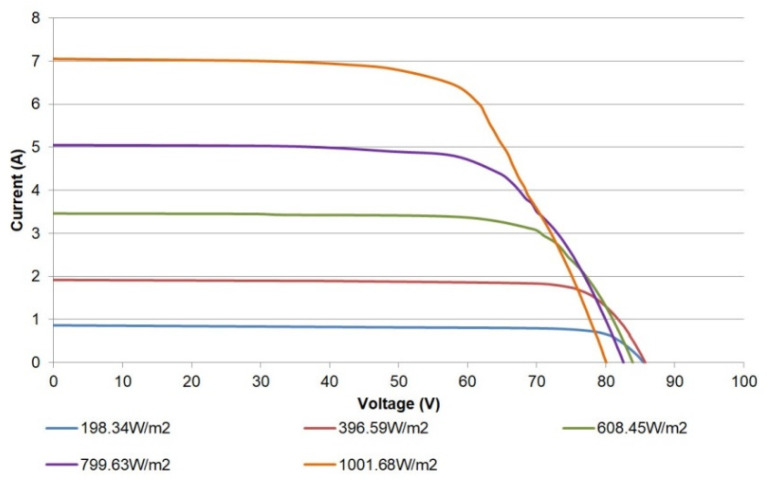
I-V curves for two PV modules connected in series.

**Figure 19:**
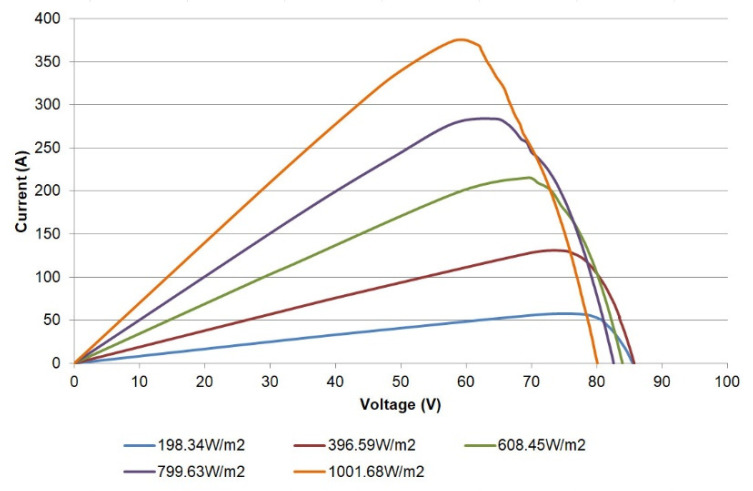
P-V curves of a pair of PV modules.

The traced I-V curves for a pair of modules connected in series are depicted in Figure 18. The shape observed in the traced curves allows diagnosing or detecting diverse effects in the PV modules, as pointed out in previous works [7,36]. In this case, the curves show a certain alteration in the inflection point and slopes, which indicate the degradation of one of the modules. Therefore, these curves serve for fault detection and diagnostics; namely, aging effects, cell cracking, hot spots, potential induced degradation, and other deterioration situations can be detected. In fact, the modules have been working for 10 years, so aging effects can be expected. Nonetheless, in-depth diagnosis and fault analyses are out of the scope of this paper. On the other hand, Figure 19 contains the traced P-V curves for the pair of modules.

**Figure 20:**
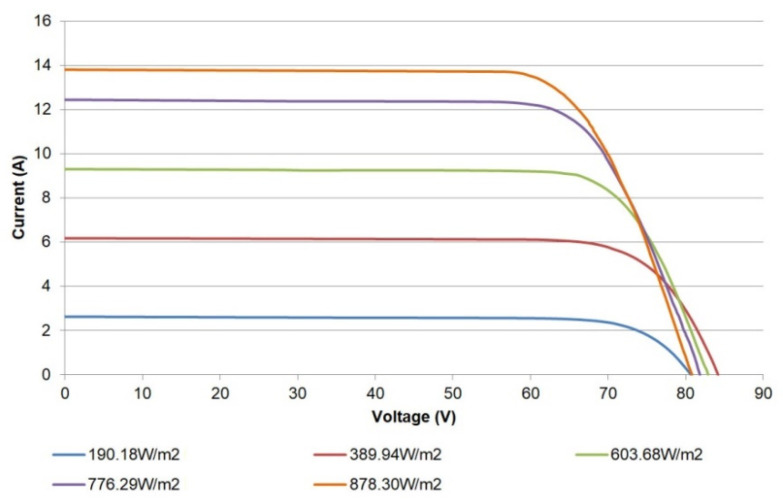
I-V curves of PV generator.

**Figure 21:**
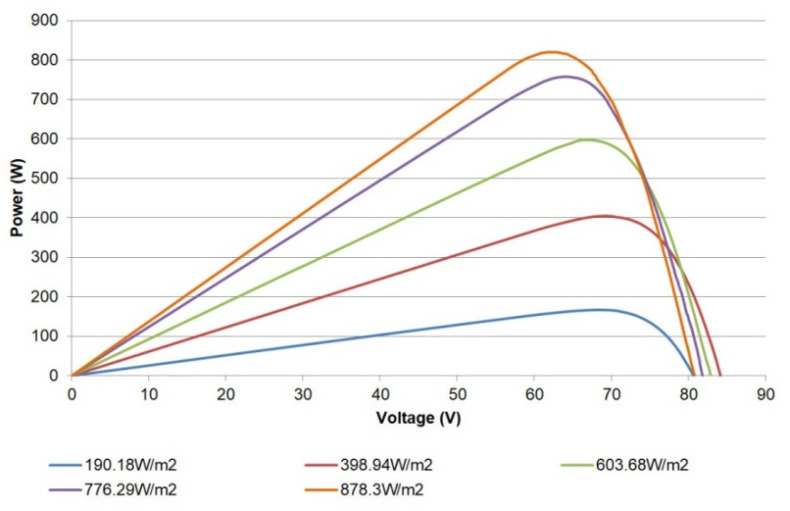
P-V curves of PV generator.

Finally, the I-V curves captured for the whole PV generator are shown in Figure 20. The corresponding P-V curves are depicted in Figure 21. As in the previous figures, the curves display the expected operation of the generator and can be applied for diagnostics purposes.

## 4. Discussion

Experimental results provide I-V curves for different electrical configurations and environmental conditions, emphasizing the suitability of the designed curve tracer.

The main strength is that the developed system is not limited to data acquisition of PV modules for I-V curves, but data recording and visualization in real-time during the characterization are also entirely approached. Indeed, once the desired conditions are programmed, the fully autonomous operation of the curve tracer is achieved without requiring the intervention of the operator.

The deployed curve tracer consists of the RPi and the associated software, whilst a PLC and a programmable load of an experimental SMG are used to validate the operation of such curve tracer. 

The computational capabilities of the microprocessor are proven to be adequate to resolve for data acquisition, storage, and visualization. It must be emphasized that none of the previous literature provides information in this regard.

Using an in-house database (mariaDB) and a web-enabled user interface (Grafana) avoids dependencies on external servers and the associated hosting or licensing costs. Hosting on one’s own databases even implies a total control of administration aspects [4].

As a proof of concept, in the reported application case, Modbus TCP and RS232 have been used. However, the curve tracer can manage virtually any communication protocol given the wide availability of libraries on the Internet. Furthermore, this ability to support many other protocols provides features such as configurability and modularity, facilitating interoperability [46].

In particular, the use of open communication protocols such as Modbus TCP together with the ability of the open-source equipment allows for the establishment of seamless data exchange. This way, proprietary equipment (PLC) is combined with the curve tracer without interoperability issues. In fact, logical connections through communication protocols enable measurement information sharing and facilitate integration in real scenarios, which constitutes a disadvantage in most of the curve tracers in the literature [5]. In this regard, the deployed system is focused on PV facilities already existent, so the coupling is made through the aforementioned open protocol. The curve tracer even makes use of already existing sensors, which is a benefit since the PV generator can be re-characterized when required without essential alterations in the electrical and communications schemes. 

Instead of using a variable resistor, capacitive load, or a power MOSFET, the proposal employs a real electronic programmable load to perform the I-V tracing. In addition, the used load is legacy equipment which does not support modern communication interfaces, so being able to manage such valuable equipment is an important advantage. In fact, IoT technologies must contribute to solve compatibility and interoperability issues with legacy devices [47,48].

Regarding economic assessments, the cost of the curve tracer is very low given the inexpensive nature of the IoT open-source equipment, which constitutes an advantage of scientific equipment based on this type of technology [49]. Namely, taking into account that all the software is free (Python, mariaDB, Grafana), only the RPi involves expenses; the overall cost is around EUR 70. Auxiliary elements such as a memory card, heatsink and fan for cooling, and power adapter are also included. 

Analyses of the retrieved I-V curves allow decision making with respect to operation and maintenance of the PV modules as well as implementing accurate models. Moreover, further experiments will include partial shading of the PV modules in order to obtain and analyze the measured I-V curves.

Thanks to the flexibility and availability of open-source equipment, the system can be customized to fulfill particular requirements in research or academic contexts. The RPi provides a large number of analogue and digital inputs, allowing the connection of additional sensors or instruments. Indeed, advances in IoT technology, both hardware and software, can be integrated in the presented system. 

Despite the obtained results, the presented system has some limitations which are now briefly described. To begin with, managing open-source technology does not imply ease of configuration when advanced functions are required. For example, programming skills and a certain expertise in communication protocols and networks are needed. In addition, the proposal does not allow online measurements of the PV modules; it is only devoted to offline characterization. For a proper data exchange, it is necessary that the automation unit (PLC or similar device) and the programmable load provide communication interfaces that the RPi can handle. It is not a probable boundary in modern devices, but for legacy equipment, it must be carefully tackled. Finally, the representation of the I-V curves requires manual data extraction from the files that Grafana stores and provides. This can be a time-consuming task when a large number of measurements have been conducted. 

## 5. Conclusions

RES are key enablers for the evolution towards a more sustainable energetic global scenario, PV technology being one of the most applied RES in microgrids. In order to characterize and study the behavior of PV modules, an I-V curve tracer based on IoT open-source technologies has been presented. Namely, software such as Python, MariaDB, and Grafana run on an RPi are responsible for automating all the required tasks: load sweep, data acquisition, data storage, communications, and visualization in real-time. An open communication protocol (Modbus TCP) has been applied to exchange information with a PLC, whilst an RS232 allows for managing a legacy programmable load. Both proprietary devices belong to a research-oriented microgrid facility and serve as proof of concept to prove the suitability of the curve tracer. 

It must be emphasized that this development is a novelty in the existing literature, addresses trends, and overcomes limitations identified in previous works, among which short-time measurements, low-cost measurement systems, low-cost communications, and IoT open-source technology can be highlighted.

Experimental results under real operating conditions are used to validate the proposal. Namely, a PV generator of 1110 W integrated into an SMG is characterized by means of the developed curve tracer.

Future research includes diagnostics and fault detection of the PV modules. Furthermore, another interesting topic deals with the development of an on-line characterization procedure using the presented system.

## Figures and Tables

**Table 1:** Table comparative of previous literature dealing with custom-made curve tracers.

References	Device	Load	Data Storage	Language	Communication
[6]	PIC	MOSFET	External (PC or smart device)	Unspecified	Bluetooth/USB
[10]	Microcontroller	MOSFET	Local database (NoSQL)	Unspecified	MQTT
[12]	PIC	Capacitive	Unspecified	Unspecified	Power line communication
[15]	Microcontroller	MOSFET	Local memory	Unspecified	USART/Bluetooth
[22,23]	DSP	Capacitive	External (PC)	Unspecified	Serial
[24]	Microcontroller	Capacitive	Unspecified	Unspecified	Unspecified
[21]	Arduino	Capacitive	External (PC)	Arduino sketch	USB
[32]	Arduino	MOSFET	External (datalogger)	Arduino sketch	No
[33]	Arduino	MOSFET	External (PC)	Arduino sketch	USB
[34]	Arduino	Resistive	Local SD card	Arduino sketch	USB
[35]	RPi	Capacitive	External cloud server	Unspecified	MQTT
[36]	RPi	MOSFET	FTP server or local SD card	Python	Ethernet
[37]	RPi	Capacitive	Unspecified	Python	Ethernet/Wi-Fi
Present work	RPi	Programmable electronic load	Local database (mariaDB)	Python	Modbus TCP

**Table 2:** Magnitudes and sensors used in the curve tracer.

Magnitude	Sensor
Current	Hall-effect sensor
Voltage	Potentiometric voltage divider
Irradiance	Pyranometer
Temperature	Pt-100

**Table 3:** Main parameters of PV modules.

Parameter	Value
Model	LDK Solar 185D-24S
Maximum power (Pmax)	185 W
Voltage at max power (Vmp)	36.9 V
Current at max power (Imp)	5.02 A
Open circuit voltage (Voc)	45.1 V
Short-circuit current (Isc)	5.48 A
Nominal voltage	24 V
Number of solar cells	72
Cell efficiency	17.77%
Module efficiency	14.49%

**Table 4:** Irradiance, temperature, and electrical parameters measured during characterization for different configurations of the PV modules.

Configuration	G (W/m^2^)	T (°C)	Voc (V)	Isc (A)	Vmp (V)	Imp (A)	Pmax (W)
Single module	195	11.46	42.23	0.84	37.38	0.74	27.07
	401	19.70	42.49	1.91	35.50	1.79	63.72
	603	26.06	42.39	3.12	34.75	2.83	98.41
	871	41.39	41.01	5.17	29.08	4.78	139.03
	1019	45.1	40.93	6.20	29.20	5.68	165.89
Two modules in series	198.34	6.44	85.49	0.86	77.55	0.74	57.23
	396.59	14.71	85.71	1.92	74.87	1.74	130.66
	608.45	28.75	83.91	3.46	69.44	3.10	215.27
	799.63	39.05	82.57	5.07	64.50	4.42	283.80
	1001.68	50.96	80.07	7.05	58.02	6.45	374.11
Whole PV generator	190.18	11.46	80.65	2.63	67.69	2.46	166.59
	389.94	19.70	84.17	6.18	69.57	5.82	404.89
	603.68	26.06	82.89	9.35	66.74	8.96	597.85
	776.29	41.39	81.84	12.45	64.06	11.83	757.94
	878.30	45.1	80.82	13.81	62.12	13.16	820.19

## Data Availability

Not applicable.

## Abbreviations

The following abbreviations are used in this manuscript:

CPU	Central Processing Unit
DSP	Digital Signal Processor
EL	Electrolyzer
FC	Fuel cell
FTP	File Transfer Protocol
GUI	Graphical User Interface
I-V	Current-Voltage
IoT	Internet of Things
MQTT	Message Queue Telemetry Transport
OTF	Outdoor Test Facility
P-V	Power-Voltage
PC	Personal Computer
PLC	Programmable Logic Controller
PV	Photovoltaic
R&D	Research and Development
RES	Renewable Energy Sources
RPi	Raspberry Pi
SMG	Smart MicroGrid
STC	Standard Test Conditions
TCP/IP	Transmission Control Protocol/Internet Protocol
USART	Universal Synchronous Asynchronous Receiver Transmitter
USB	Universal Serial Bus

## References

[1] Gimeno-Sales F.J., Orts-Grau S., Escribá-Aparisi A., González-Altozano P., Balbastre-Peralta I., Martínez-Márquez C.I., Gasque M., Seguí-Chilet S. (2020). PV Monitoring System for a Water Pumping Scheme with a Lithium-Ion Battery Using Free Open-Source Software and IoT Technologies. Sustainability.

[2] Ansari S., Ayob A., Lipu M.S.H., Saad M.H.M., Hussain A. (2021). A Review of Monitoring Technologies for Solar PV Systems Using Data Processing Modules and Transmission Protocols: Progress, Challenges and Prospects. Sustainability.

[3] Gielen D., Boshell F., Saygin D., Bazilian M.D., Wagner N., Gorini R. (2019). The role of renewable energy in the global energy transformation. Energy Strateg. Rev..

[4] Portalo J.M., González I., Calderón A.J. (2021). Monitoring System for Tracking a PV Generator in an Experimental Smart Microgrid: An Open-Source Solution. Sustainability.

[5] Dupont I.M., Carvalho P.C.M., Jucá S.C.S., Neto J.S.P. (2019). Novel methodology for detecting non-ideal operating conditions for grid-connected photovoltaic plants using Internet of Things architecture. Energy Convers. Manag..

[6] Vega A., Valiño V., Conde E., Ramos A., Reina P. (2019). Double sweep tracer for I-V curves characterization and continuous monitoring of photovoltaic facilities. Sol. Energy.

[7] Morales-Aragonés J.I., Dávila-Sacoto M., González L.G., Alonso-Gómez V., Gallardo-Saavedra S., Hernández-Callejo L. (2021). A Review of I–V Tracers for Photovoltaic Modules: Topologies and Challenges. Electronics.

[8] Melo G.C.G.D., Torres I.C., Araújo Í.B.Q.D., Brito D.B., Barboza E.D.A. (2021). A Low-Cost IoT System for Real-Time Monitoring of Climatic Variables and Photovoltaic Generation for Smart Grid Application. Sensors.

[9] Quansah D.A., Adaramola M.S., Takyi G., Edwin I.A. (2017). Reliability and Degradation of Solar PV Modules—Case Study of 19-Year-Old Polycrystalline Modules in Ghana. Technologies.

[10] Papageorgas P., Piromalis D., Valavanis T., Kambasis S., Iliopoulou T., Vokas G. (2015). A low-cost and fast PV I-V curve tracer based on an open source platform with M2M communication capabilities for preventive monitoring. Energy Procedia.

[11] Zhu Y., Xiao W. (2020). A comprehensive review of topologies for photovoltaic I–V curve tracer. Sol. Energy.

[12] Ortega E., Aranguren G., Jimeno J.C. (2019). New monitoring method to characterize individual modules in large photovoltaic systems. Sol. Energy.

[13] Toledo C., Serrano-Lujan L., Abad J., Lampitelli A., Urbina A. (2019). Measurement of Thermal and Electrical Parameters in Photovoltaic Systems for Predictive and Cross-Correlated Monitorization. Energies.

[14] Sarikh S., Raoufi M., Bennouna A., Ikken B. (2021). Characteristic curve diagnosis based on fuzzy classification for a reliable photovoltaic fault monitoring. Sustain. Energy Technol. Assess..

[15] Morales-Aragonés J.I., Gallardo-Saavedra S., Alonso-Gómez V., Sánchez-Pacheco F.J., González M.A., Martínez O., Muñoz-García M.A., Alonso-García M.d.C., Hernández-Callejo L. (2021). Low-Cost Electronics for Online I-V Tracing at Photovoltaic Module Level: Development of Two Strategies and Comparison between Them. Electronics.

[16] Mellit A., Kalogirou S. (2021). Artificial intelligence and internet of things to improve efficacy of diagnosis and remote sensing of solar photovoltaic systems: Challenges, recommendations and future directions. Renew. Sustain. Energy Rev..

[17] Anani N., Ibrahim H. (2020). Adjusting the Single-Diode Model Parameters of a Photovoltaic Module with Irradiance and Temperature. Energies.

[18] Web of Commercial Curve Tracer of Manufacturer Atecorp. https://www.atecorp.com/products/tritec/tri-ka-iv.

[19] Web of Commercial Curve Tracer of Manufacturer LabX. https://www.labx.com/item/daystar-energy-engineering-ds-100c-photovoltaic-iv/LV41115631.

[20] Web of Commercial Curve Tracer of Manufacturer Ht-Instruments. https://www.ht-instruments.com/es-es/productos/instrumentacion-fotovoltaica/medidores-curva-i-v/i-v500w/.

[21] Montes-Romero J., Piliougine M., Muñoz J.V., Fernández E.F., De la Casa J. (2017). Photovoltaic Device Performance Evaluation Using an Open-Hardware System and Standard Calibrated Laboratory Instruments. Energies.

[22] Chen Z., Lin W., Wu L., Long C., Lin P., Cheng S. (2017). A capacitor based fast I-V characteristics tester for photovoltaic arrays. Energy Procedia.

[23] Chen Z., Lin Y., Wu L., Cheng S., Lin P. (2020). Development of a capacitor charging based quick I-V curve tracer with automatic parameter extraction for photovoltaic arrays. Energy Convers. Manag..

[24] Cáceres M., Firman A., Montes-Romero J., González Mayans A.R., Vera L.H., Fernández F.E., de la Casa Higueras J. (2020). Low-Cost I–V Tracer for PV Modules under Real Operating Conditions. Energies.

[25] González I., Calderón A.J. (2019). Integration of open source hardware Arduino platform in automation systems applied to Smart Grids/Micro-Grids. Sustain. Energy Technol. Assess..

[26] Fuentes M., Vivar M., Burgos J.M., Aguilera J., Vacas J.A. (2014). Design of an accurate, low-cost autonomous data logger for PV system monitoring using Arduino™ that complies with IEC standards. Sol. Energy Mater. Sol. Cells.

[27] Vargas-Salgado C., Aguila-Leon J., Chiñas-Palacios C., Hurtado-Pérez E. (2019). Low-cost web-based Supervisory Control and Data Acquisition system for a microgrid testbed: A case study in design and implementation for academic and research applications. Heliyon.

[28] González I., Calderón A.J., Portalo J.M. (2021). Innovative Multi-Layered Architecture for Heterogeneous Automation and Monitoring Systems: Application Case of a Photovoltaic Smart Microgrid. Sustainability.

[29] Paredes-Parra J.M., Mateo-Aroca A., Silvente-Niñirola G., Bueso M.C., Molina-García Á. (2018). PV module monitoring system based on low-cost solutions: Wireless raspberry application and assessment. Energies.

[30] Pereira R.I.S., Dupont I.M., Carvalho P.C.M., Jucá S.C.S. (2018). IoT embedded linux system based on Raspberry Pi applied to real-time cloud monitoring of a decentralized photovoltaic plant. Measurement.

[31] IV Swinger Documentation. https://github.com/csatt/IV_Swinger.

[32] Willoughby A.A., Osinowo M.O. (2018). Development of an electronic load I-V curve tracer to investigate the impact of Harmattan aerosol loading on PV module pern2tkformance in southwest Nigeria. Sol. Energy.

[33] Amiry H., Benhmida M., Bendaoud R., Hajjaj C., Bounouar S., Yadir S., Rais K., Sidki M. (2018). Design and implementation of a photovoltaic I-V curve tracer: Solar modules characterization under real operating conditions. Energy Convers. Manag..

[34] Pachauri R.K., Mahela O.P., Khan B., Kumar A., Agarwal S., Alhelou H.H., Bai J. (2021). Development of arduino assisted data acquisition system for solar photovoltaic array characterization under partial shading conditions. Comput. Electr. Eng..

[35] Shapsough S., Takrouri M., Dhaouadi R., Zualkernan I. (2020). An IoT-based remote IV tracing system for analysis of city-wide solar power facilities. Sustain. Cities Soc..

[36] Sarikh S., Raoufi M., Bennouna A., Benlarabi A., Ikken B. (2020). Implementation of a plug and play I-V curve tracer dedicated to characterization and diagnosis of PV modules under real operating conditions. Energy Convers. Manag..

[37] Sayyad J., Nasikkar P., Singh A.P., Ozana S. (2021). Capacitive Load-Based Smart OTF for High Power Rated SPV Module. Energies.

[38] Cotfas P.A., Cotfas D.T. (2020). Comprehensive Review of Methods and Instruments for Photovoltaic–Thermoelectric Generator Hybrid System Characterization. Energies.

[39] Torres-Moreno J.L., Gimenez-Fernandez A., Perez-Garcia M., Rodriguez F. (2018). Energy Management Strategy for Micro-Grids with PV-Battery Systems and Electric Vehicles. Energies.

[40] Elkazaz M., Sumner M., Pholboon S., Davies R., Thomas D. (2020). Performance Assessment of an Energy Management System for a Home Microgrid with PV Generation. Energies.

[41] Yerasimou Y., Kynigos M., Efthymiou V., Georghiou G.E. (2021). Design of a Smart Nanogrid for Increasing Energy Efficiency of Buildings. Energies.

[42] Jaloudi S. (2019). Communication Protocols of an Industrial Internet of Things Environment: A Comparative Study. Future Internet.

[43] Grafana Dashboard for Monitoring System Metrics Based on Telegraf. https://grafana.com/grafana/dashboards/928.

[44] Pindado S., Cubas J., Roibás-Millán E., Bugallo-Siegel F., Sorribes-Palmer F. (2018). Assessment of Explicit Models for Different Photovoltaic Technologies. Energies.

[45] Álvarez J.M., Alfonso-Corcuera D., Roibás-Millán E., Cubas J., Cubero-Estalrrich J., Gonzalez-Estrada A., Jado-Puente R., Sanabria-Pinzón M., Pindado S. (2021). Analytical Modeling of Current-Voltage Photovoltaic Performance: An Easy Approach to Solar Panel Behavior. Appl. Sci..

[46] Hosseinzadeh N., Al Maashri A., Tarhuni N., Elhaffar A., Al-Hinai A. (2021). A Real-Time Monitoring Platform for Distributed Energy Resources in a Microgrid—Pilot Study in Oman. Electronics.

[47] Akerman M., Fast-Berglund A., Ekered S. (2016). Interoperability for a dynamic assembly system. Procedia CIRP.

[48] Nițulescu I.-V., Korodi A. (2020). Supervisory Control and Data Acquisition Approach in Node-RED: Application and Discussions. IoT.

[49] Pearce J.M. (2020). Economic savings for scientific free and open source technology: A review. HardwareX.

